# Supplementary material for: Deregulated Clusterin as a Marker of Bone Fragility: New Insights into the Pathophysiology of Osteoporosis
Source: Genes (Basel). 2022 Apr 7;13(4):652. doi: 10.3390/genes13040652 (PMC9024451; doi:10.3390/genes13040652)
Supplement: Supplementary file 1 [file genes-13-00652-s001.zip › genes-1655035-supplementary.pdf]

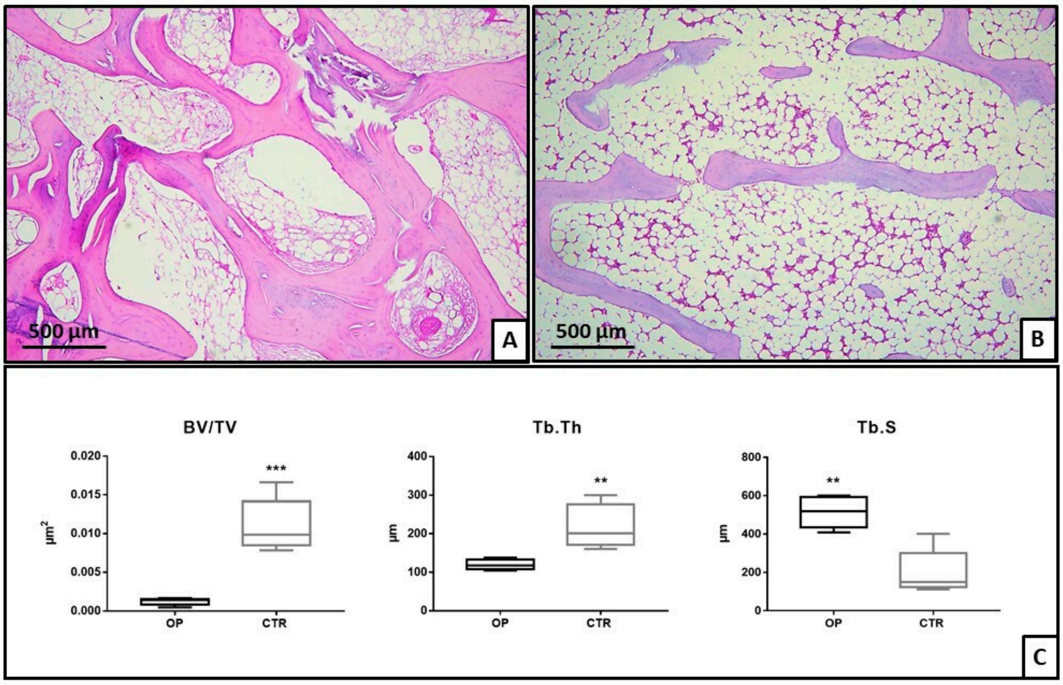

Figure S1. Haematoxylin and Eosin (H&E) sections of bone head biopsies and bone morphometric analysis. The analysis of conventional bone morphometric parameters, such as bone volume (BV/TV), trabecular thickness (Tb.Th) and trabecular separations (Tb.S) was carried out in haematoxylin and eosin (H&E) femoral head bone sections. Ten microscopic images, randomly selected, were evaluated for each biopsy sample. Images were acquired at 4x magnification using a Nikon upright microscope ECLIPSE CiS/(Nikon Corporation, Tokyo, Japan) connected to a Nikon digital camera. Image analysis was performed using NIS-Elements software (5.30.01; Laboratory Imaging, Prague, Czech Republic). (**A**) The bone tissue of CTR patients appeared to be characterized by a well-preserved trabecular network; (**B**) in contrast, the femoral head bone tissue of OP patients showed a marked decrease in trabecular thickness, resulting in an increase in inter-trabecular space and a decrease in bone volume. (**C**) The histomorphometric analysis showed a significant difference between the two experimental groups for each parameter: it was observed a significant reduction of bone volume (BV/TV) and trabecular thickness (Tb.Th) in the OP group compared to CTR subjects (OP, BV/TV: 0.001236 μm^2, Tb.Th: 120.1 µm *vs* CTR, BV/TV: 0.011026 μm^2, Tb.Th: 218.8 µm; ***$p<0.001$, **$p<0.01$), and an increment of Tb.S values in OP group compared to CTR group (OP, Tb.S: 515.5 µm *vs* CTR, Tb.S: 199.7 µm; **$p<0.01$). (H&E 4x, scale bar represents 500 µm).

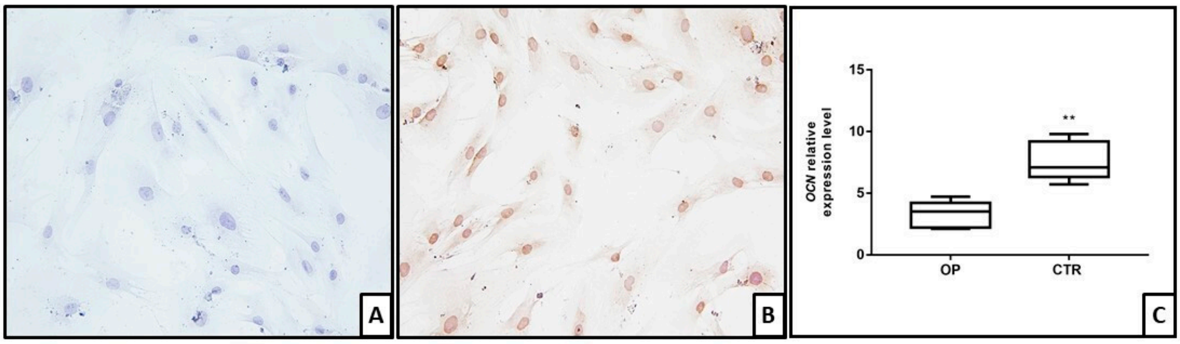

Figure S2. Characterization of primary osteoblast cultures. The cells were plated in 24 well at a concentration of 30,000 cells/well. After 2 days, the medium was removed and cells were fixed in formalin 10% for 5 min and stored at 4 ◦C in PBS1X, to perform immunocytochemistry (ICC) analysis. After fixation, cell cultures were pretreated with Ethylenediaminetetraacetic acid (EDTA) citrate pH 7.8 for 20 min at 95 ◦C. Then, cells were incubated with rabbit anti-RUNX2 antibody (#12556 Cell Signalling Technology). After 1 hour and three washes in TBS1X/0,05% Triton X-100, cells were incubated for 15 minutes at room temperature with secondary antibody (ScyTek Laboratories, UltraTek Anti-Polyvalent Biotinylated Antibody). After repeating three washes as above, cells were incubated with Streptavidine solution (ScyTek Laboratories, UltraTek HRP) for 15 minutes. Finally, after the additional three washes, cells were incubated with 3,3′-Diaminobenzidine (DAB) for signal detection, and counterstained with haematoxylin. A negative control of the reaction was used. (**A**) Human osteoblasts primary culture used as negative control; (**B**) human osteoblasts primary culture incubated with primary antibody anti-RUNX2. (**C**) The relative difference of *OCN* gene expression between OP and CTR subjects was calculated using $2^{-\Delta\Delta C_T}$ method and normalized to *B2M* and *β-actin* levels as the internal control.

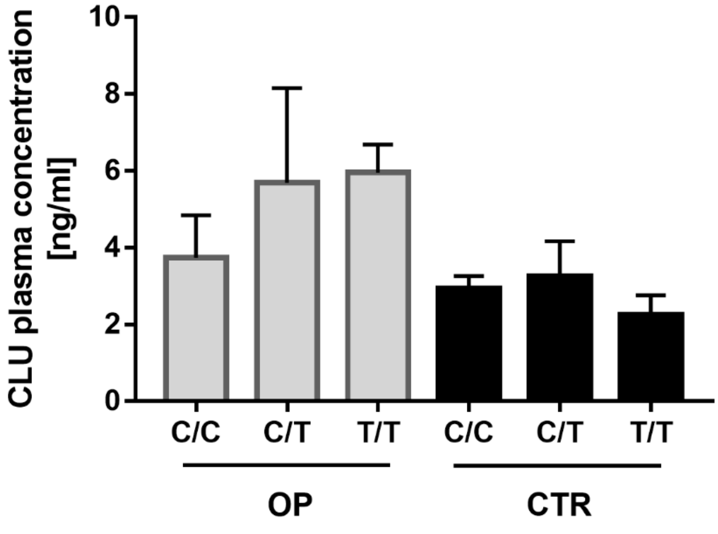

Figure S3. Expression level of CLU in OP and CTR individuals with different rs11136000 genotypes. *CLU* expression in OP patients with C/C ($n = 10$), C/T ($n = 18$) and T7T ($n = 2$) genotypes (C/C *vs* C/T $p = 0.192$; C/C *vs* T/T $p = 0.373$; C/T *vs* T/T, $p = 0.983$). *CLU* expression CTR subjects with genotype C/C ($n = 11$), C/T ($n = 15$) and T/T ($n = 4$), respectively (C/C *vs* C/T $p = 0.753$; C/C *vs* T/T $p = 0.452$; C/T *vs* T/T, $p = 0.254$). Statistical differences between groups were analysed for significance using one-way ANOVA test.
